# Commentary: Definitely maybe: can unconscious processes perform the same functions as conscious processes?

**DOI:** 10.3389/fpsyg.2017.01230

**Published:** 2017-08-04

**Authors:** Ariel Goldstein, Ran R. Hassin

**Affiliations:** ^1^Cognitive Science Department, Hebrew University Jerusalem, Israel; ^2^Psychology Department, Hebrew University Jerusalem, Israel; ^3^Cognitive Science Department, Center for the Study of Rationality, Hebrew University Jerusalem, Israel

**Keywords:** nonconscious, high level cognition, CFS, implicit, subliminal

Through critically examining Hassin's paper “Yes It Can” (YIC; 2013) Hesselmann and Moors (H&M; 2015) suggest that existing data support a more moderate, skeptical view than that suggested by Hassin. The thing we like the most about H&M's paper is the view it proposes: “Definitely Maybe.” To the best of our understanding, the view that H&M suggest to those of us interested in non-conscious high-level cognitive processes—open minded skepticism—is definitely an improvement on the more traditional “No It Can't” (e.g., Newell and Shanks, 2014). Definitely Maybe leaves a door open, conceptually and empirically.

The argument Hassin made in YIC is simple. He suggested that the capacity limitations of our conscious processes, as well as evolutionary considerations, make it reasonable to suspect that non-conscious processes can carry out every fundamental high-level function that conscious processes can perform. He then went on to review the literature on a number of functions that were traditionally associated with consciousness, and showed that there are already data to suggest that they can occur non-consciously (see also Hassin and Sklar, 2014). The functions that Hassin reviewed in YIC were cognitive control and executive functions, pursuing goals, and information broadcasting and reasoning. Each section reviewed multiple papers that were representative, but far from exhaustive. The function of the literature review was simple: “to *illustrate* [emphasis added] YIC in fundamental, high-level cognitive functions” (p. 196). The review rendered the argument for YIC more plausible simply by pointing out that many data exist to support it.

H&M offer three main criticisms of YIC that have to do with the supporting evidence reviewed in the paper. We thank them very much for making these comments, and for the way they make them. We believe science is a social endeavor that advances by exchanges of this sort. We will address each of their points below.

## H&M's review

In their first section H&M argue that Hassin's review of the literature was selective, and many of their points are well taken. Note, however, that much of the data that was left out of YIC was supportive: there is so much additional data out there about non-conscious high-level cognitive and motivational functions that Hassin had to limit himself in certain ways (see Bargh and Morsella, 2008; Bargh et al., 2012; for other relevant reviews and overviews see Kouider and Dehaene, 2007; Van den Bussche et al., 2009 as well as Kahneman, 2011; Dehaene, 2014).

Ironically, yet naturally, H&M's review of YIC is…selective too. In their first section, they raise concerns about a small subset of the findings Hassin reviewed. They ignore most of the papers reviewed in YIC, in most of its sections. Just to Illustrate: none of the investigations of non-conscious cognitive control were examined or questioned (for a review of more of those see Hassin and Sklar, 2014). In the reasoning section, they selectively focus on issues of replicability and limitations of Unconscious Thought Theory (Dijksterhuis, 2006; Dijksterhuis and Nordgren, 2008; but see also the meta-analysis in Strick et al., 2011; Nieuwenstein et al., 2015), and flag priming in the U.S. (but see Ferguson et al., 2013), but do not discuss other sections such as the vast literatures on inferences, insights, and others.

Are there question marks about some of the findings, including our own? Yes, and we thank H&M for pointing out those that were erroneously left out of the original paper and those that have become public since. Will there be more question marks in the future? Definitely. After all, this is how science progresses and corrects itself. But are the challenges so far detrimental to YIC? We do not think so. In fact, as questions were being raised about a small subset of the findings in the original review, many new findings were published too.

To take a few examples, the idea that humans can non-consciously read multiword expressions (Sklar et al., 2012) was supported by new findings and different paradigms (Armstrong and Dienes, 2013, 2014; van Gaal et al., 2014; Axelrod et al., 2015; Saxe personal communication). In these papers, multiple-word expressions were presented subliminally, and behavioral and neurological evidence supported the claim that participants were able to read and understand them. New findings extend the previous literature on non-conscious executive function and implicit effects of working memory (Gayet et al., 2013; Dutta et al., 2014), as well as implicit emotion regulation (Wang and Li, 2017). Non-conscious information integration, yet another function that was once perceived as requiring consciousness, was demonstrated in new ways (Alsius and Munhall, 2013; Faivre et al., 2014; Mudrik et al., 2014; Fahrenfort et al., 2017; Hung et al., 2017; but see Moors et al., 2016); various laboratories have shown that information can be non-consciously integrated across time by demonstrating motion perception with subliminal stimuli (Kaunitz et al., 2011; Kimura et al., 2012; Faivre and Koch, 2014a,b; Salomon et al., 2016; Moors et al., 2017); it has been shown that narratives can be extracted from subliminal stimuli (Kawakami and Yoshida, 2015); and, relatedly, Moors et al. (2017) have shown that causality is inferred and used non-consciously too (non-casual events become conscious after causal events). Lastly, priming bankers with their banker's identity leads to increased dishonesty (Cohn et al., 2014).

Again, this list is very selective, and does not begin to cover all relevant findings since the publication of YIC. Taken together, these findings, from various laboratories and using different paradigms, significantly challenge the modal view of unconscious processes vis-à-vis reading, and support the main claim of YIC.

## Priming in social and cognitive psychology

As Hassin pointed out in YIC (see also Doyen et al., 2014), the processes that our minds can do with stimuli that we do not consciously perceive are a (small?) subset of the processes that our minds can do without awareness. This is the distinction between the downstream effects of subliminal perception and the effects of unconscious cognition (Bargh and Morsella, 2008). Anyone interested in the cognitive unconscious—or in human consciousness—should be interested in both types of processes.

It should not come as a surprise, then, that we do not agree with H&M's argument, according to which the shift from a focus on unconscious perception (i.e., subliminal perception) to unawareness of processes and their effects (i.e., unconscious cognition) is “problematic” (p. 2). It is anything but, and it is a necessary step if one wants to understand the contribution of unconscious processes to human existence. Studying unconscious processes solely through subliminal perception is akin to studying gender differences solely through differences in physical appearance, or studying human memory solely by examining memorization of lists. Yes, there are differences in appearances, and important insights to be gained from studying list memorization, but there is much more to gender differences and memory than that.

H&M go on to contrast the views of Doyen et al. (2014) and Hassin vis–à-vis the differences between the two traditions of studying the unconscious. We wish to note that the views are not mutually exclusive. While some of the differences are likely to be the result of the factors identified by Doyen et al., others probably have to do with motivation, practice, and ability, the factors identified by Hassin. Traditional views of automaticity confounded unconsciousness with involuntariness. Bargh (1994) suggested that these characteristics do not necessarily go hand in hand. We take this argument one step further and suggest that unconscious processes are more likely to happen with stimuli and ideas we care deeply about than with those we care less about (Hassin, 2013). The reader is invited to think about the number of times she had new insights and surprising thoughts about issues she didn't care about vs. those she really cared about.

H&M suggest that in social psychology, where some of the major advances in understanding the human unconscious have occurred in recent decades, “absence of awareness is often assumed rather than tested, and when tests are conducted, they are below the standards widely used in cognitive psychology” (p. 2). We tend to agree (noting, of course, that the qualifier OFTEN is crucial), and we definitely join H&M's call for the adoption of ever more sophisticated ways of measuring and assessing awareness—its stimuli, effects, and processes. We wish to highlight two points here, though. One, that the standards in cognitive psychology are less clear than one may want them to be. This literature is marred with disagreements.

Secondly, it is important to highlight that in both cognitive and social psychology, the role of consciousness is often assumed, and hardly ever put for to a rigorous test (Hassin and Milyavsky, 2014). In fact, even if data convincingly show that a function F cannot happen non-consciously, these data are mute vis. a vis. causality, a point that seems to be overlooked by many. More specifically, assume that we become convinced that a function F is not executed when the process is non-conscious. This is a correlational observation, and it doesn't mean that consciousness has a causal role in allowing F. There might be a factor that brings about consciousness and allows F to happen; or there might be a factor that tends to co-occur with consciousness, that allows F to happen. When we look back at the literature, for example, it is quite clear that for many years we confounded prime duration with consciousness. Subliminal stimuli had to be very short. As CFS experiments have shown, longer duration subliminal stimuli seem to have more effects than shorter duration subliminal stimuli (but see Barbot and Kouider, 2012), so some functions we attributed to consciousness should be attributed to duration.

## On optimism and CFS

CFS is a relatively new methodology, and as such there are many welcomed debates surrounding it. H&M discuss papers that suggest limitations in non-conscious processing under CFS, and how to measuring measure awareness under CFS is also a question in active debate (Gelbard-Sagiv et al., 2016). In their third section H&M build on these findings to suggest that enthusiasm about CFS as a tool for unconscious research is “premature and farfetched.” It is definitely a possibility. CFS is young (only 10 years old; Tsuchiya and Koch, 2005), and only future data will tell.

But there are two reasons for cautious optimism. The format of this reply is short, but let us just note that even if it turns out that there is partial low-level awareness in CFS that does not mean that the process under consideration is conscious. Knowing that a word is written in blue (vs. red) ink, doesn't mean that you can consciously read the word, which is the question we should focus on when we examine reading (for example). Secondly, CFS has been used to show non-conscious semantic integration (Mudrik et al., 2011, 2014; but see Moors et al., 2016), non-conscious working memory (Pan et al., 2012; Gayet et al., 2013), non-conscious multisensory integration (Alsius and Munhall, 2013; Faivre et al., 2014), non-conscious emotion recognition (Capitão et al., 2014), non-conscious reading (Costello et al., 2009; Yang and Yeh, 2011; Zabelina et al., 2013), non-conscious emotion perception (Yang et al., 2007; Almeida et al., 2013; Troiani and Schultz, 2013) and non-conscious expectations (Stein and Peelen, 2015), amongst other functions. For such a young technique, these results are very encouraging.

## Between maybe and yes

Why do lay people and scientists alike have the strong intuition that there simply must be cognitive functions that are made possible only by the kind of consciousness we have? We don't know, but we suspect it has to do with the search for the one thing that makes us, human beings, special, so that we stand out from the animal kingdom. It has to do with the desire, so beautifully identified by Dan Gilbert, “to publish a book, a chapter, or at least an article that contains this sentence: The human being is the only animal that….” (Stumbling on Happiness, p. 3) (Gilbert, 2006). And indeed, there is a correlation that is hard to miss: we (think we) are very special in the animal world and we (think we) have consciousness that no other animal has. Hence, goes the argument, there must be functions that consciousness allows for, and that make us so special.

This intuition is misleading in at least two respects. First, it is unclear whether the argument's assumptions are true. Second, and more importantly, it is logically flawed. Even if we are very special in the ways we think we are, and even if we have consciousness that no other animal has, this does not mean that the latter causes the former. Ulric Neisser, for example, had a radically different suggestion, one to which we are very sympathetic: “our hypothesis thus leads us to the radical suggestion that the critical difference between the thinking of humans and of lower animals lies not in the existence of consciousness but in the capacity for complex processes outside it” (Neisser, 1963, p. 10).

One of the implications of YIC is that the search for a holy grail—the function that only consciousness can do—is the wrong way to go. Understanding what it means to be human would be better achieved by understanding the similarities and differences between conscious and unconscious pursuit of the same functions, how conscious and unconscious functions work together and orchestrate human cognition, and how unconscious processes pursue functions that only they can pursue.

## Summary

We thank H&M for drawing our attention to shortcomings of our previous paper, and for the opportunity to exchange ideas, and we look forward to discovering more about consciousness and the human unconscious. While Definitely Maybe is definitely an improvement on the more traditional No It Can't, we still believe that Yes It Can is a more plausible alternative that directs our science to a fruitful agenda.

## Author contributions

All authors listed have made a substantial, direct and intellectual contribution to the work, and approved it for publication.

### Conflict of interest statement

The authors declare that the research was conducted in the absence of any commercial or financial relationships that could be construed as a potential conflict of interest.
